# Design and validation of a laboratory-developed diagnostic assay for monkeypox virus

**DOI:** 10.1007/s11262-023-02024-9

**Published:** 2023-08-17

**Authors:** Nikola Sklenovská, Mandy Bloemen, Valentijn Vergote, Anne-Sophie Logist, Bert Vanmechelen, Lies Laenen, Emmanuel André, Jean-Jacques Muyembe-Tamfum, Elke Wollants, Marc Van Ranst, Piet Maes, Tony Wawina-Bokalanga

**Affiliations:** 1 Laboratory of Clinical and Epidemiological Virology, Department of Microbiology, Immunology and Transplantation, Rega Institute, KU Leuven, Leuven, Belgium; 2 Department of Laboratory Medicine, UZ Leuven University Hospital, Leuven, Belgium; 3 Laboratory of Clinical Microbiology, Department of Microbiology, Immunology and Transplantation, Rega Institute, KU Leuven, Leuven, Belgium; 4 Institut National de Recherche Biomédicale, Kinshasa, Democratic Republic of the Congo

**Keywords:** Monkeypox virus, Mpox, Real-time PCR, Diagnosis

## Abstract

Mpox is a viral zoonosis with endemic circulation in animals and humans in some West and Central African countries. The disease was imported a few times in the past to countries outside the African continent through infected animals or travelers, one of which resulted in an unprecedented global outbreak sustained by human-to-human transmission in 2022. Although timely and reliable diagnosis is a cornerstone of any disease control, availability of accurate diagnostic assays and comparative performance studies of diagnostic assays remains limited despite of the long-known identification of monkeypox virus (MPXV) as a human pathogen since 1970. We laboratory-developed a real-time PCR test (LDT) and evaluated its performance against the commercial TaqMan™ Monkeypox Virus Microbe Detection Assay (Applied Biosystems, Cat A50137). The limit of detection of the LDT was established at 1.2 genome copies/ml. The sensitivity and specificity of both assays were 99.14% and 100%, respectively, and both are capable of detecting both clade I and clade II of MPXV. Our results demonstrate the validity and accuracy of the LDT for confirmation of MPXV infection from lesion swabs samples.

## Introduction

Mpox is a viral zoonotic disease, caused by monkeypox virus (MPXV), occurring historically in countries of West and Central Africa where the animal reservoir(s) is present; however, it was reported globally as part of a sustained human-to-human transmission from May 2022 [1]. The most prominent symptom of mpox is vesico-pustular rash which can lead to confusion with other rash diseases, including chickenpox, herpes simplex, disseminated herpes zoster, syphilis, measles, scabies, bacterial skin infection, and others, and need to be considered during differential clinical diagnosis of suspected mpox patients. In African countries, mpox is commonly confused with chickenpox [2], while mpox in non-African countries during the 2022 outbreak was sometimes clinically misdiagnosed with sexually transmitted infections, like herpes simplex or syphilis because patients often presented with genital lesions [3]. Therefore, accurate laboratory confirmation of MPXV infection is needed to diagnose and provide optimal clinical care to mpox patients. It is also important in order to identify, follow up, and manage contacts of mpox cases to prevent further transmission and to tailor effective control and prevention measures. Mpox laboratory confirmation relies on nucleic acid amplification tests (e.g., real-time PCR) on skin lesion material or in the absence of skin lesions on the oropharyngeal, anal, or rectal swab sample [4]. Two clades of the MPXV exist—clade I circulating mainly in the Congo Basin and historically causing more severe disease and clade II found historically mainly in West Africa, however, was exported multiple times in the past and it also caused the current global multi-country mpox outbreak [5].

Before the current multi-country outbreak affecting mainly countries of the Southern hemisphere, there was no commercial laboratory test available for MPXV which would be evaluated for clinical performance. MPXV confirmation mainly relied on the in-house laboratory-developed test (LDT), however even those were often not available in countries with known MPXV circulation in animals and human. This had an important negative impact on surveillance and thus to our understanding of the mpox burden. Geographical expansion of MPXV circulation has increased the demand for diagnostics and prompted rapid development of commercial kits [6, 7].

We have developed and validated a real-time PCR test for MPXV confirmation and this LDT was successfully used for diagnosis of mpox-suspected persons in KU Leuven, Belgium in the initial stage of the mpox outbreak in 2022. Here, we report the details of the LDT design and validation process and illustrate its analytical and clinical validity.

## Materials and methods

### Samples

*MPXV clade I samples:* Five MPXV-positive DNA samples collected in 2016 in the Democratic Republic of the Congo (DRC) were kindly provided by the National Institute of Biomedical Research (Institut National de Recherche Biomédicale; INRB), Kinshasa (DRC). These DNA samples were extracted locally at INRB from skin lesions of MPXV-infected patients. *MPXV clade II samples:* Viral DNA from the lesions swab samples of 62 MPXV-patients was extracted on the KingFisher™ Flex system (ThermoFisher) using the MagMax viral pathogen II nucleic acid isolation kit. *Varicella zoster virus (VZV) samples:* Viral DNA from lesion swab samples of 16 VZV-confirmed patients was extracted using the MagMax viral pathogen II nucleic acid isolation kit for KingFisher™ Flex system (Thermo Fisher). *Herpes simplex virus 2* (*HSV-2) samples:* Viral DNA from lesion swab samples of 2 HSV-2 confirmed patients was extracted using the MagMax viral pathogen II nucleic acid isolation kit for the KingFisher™ Flex system (ThermoFisher). The MPXV Clade II, VZV, and HSV-2 samples were collected in UZ Leuven (Belgium) in May 2022 from mpox-suspected patients where they were also confirmed.

### Design of the MPXV laboratory-developed test

#### Primers and probe

Selection of MPXV isolate genomes from both Clade I and II were retrieved from GenBank (accession numbers KJ642619.1, KJ642618.1, KJ642613.1, KP849469, KJ642617, DQ011157.1) and aligned using MEGA7 [8]. A conserved MPXV region, the open reading frame (ORF) O2L (662 bp in length), was selected as template for primer and probe design. The primers and probes were designed using the Integrated DNA Technologies PrimerQuest™ tool [9]. The melting temperature (Tm) of the primers was selected between 58 and 60 °C, while the Tm of the probes was selected 7°–10° higher to assure consistent probe binding before polymerization would occur. The oligonucleotides were checked for self-complementarity using the online tools Oligo Calc [10]. The MPXV probe was linked with 6-FAM as a dye at the 5ʹ end and ZEN-IBFQ (ZEN-Iowa black fluorescent quencher) at the 3ʹ end, with a maximum emission wavelength of 518 nm (Table 1). The primers and probe were purchased from Integrated DNA Technologies (Belgium). A BLAST search against the database *nucleotide collection (nr/nt)* was conducted using *Blastn* (Optimize for somewhat similar sequences) on February 24, 2023 for each primer and probe separately. The *organism* was set to monkeypox virus (taxid: 10244).

**Table 1:** Primers and probe for MPXV

MPXV viral ORF	Type	Oligonucleotide sequence
O2L	Probe	6-FAM-5ʹ-ACCGGTAATCTTGTCGAGGAGGACA-3ʹ-ZEN-IBFQ
	Primer forward	5ʹ-TAGTGAGTTCGGCGACAAAG-3ʹ
	Primer reverse	5ʹ-GTATCGCATCTCTCGGGTATTC-3ʹ

#### Quantitative PCR

qPCR with TaqMan Fast Virus 1-Step Master Mix (Applied Biosystems, USA) was performed in a 20-µL reaction mixture containing 5-µL DNA template, 5-µL 4× TaqMan Fast Virus 1-Step Master Mix, 250-nM (final concentration; FC) probe, 900-nM (FC) forward primer, 900-nM (FC) reverse primer, and water (Sigma-Aldrich, USA) up to 20 µL. The reactions were performed under the following conditions: 20 s at 95 °C and 45 cycles of 3 s at 95 °C and 30 s at 60 °C (Fast Mode). The reactions were run on a Quantstudio™ 7 Flex Real-Time PCR system (Applied Biosystems, Thermo Fisher).

#### Confirmation through Sanger sequencing

The PCR products were purified using the ExoSAP-IT kit (Applied Biosystems, USA) and sent to the Macrogen facilities (The Netherlands) for Sanger sequencing. Obtained sequences were analyzed and corrected using SeqMan software, version 7.0.0. A BLAST search against the database *nucleotide collection (nr/nt)* was conducted using *Blastn* (Optimize for somewhat similar sequences) on February 15, 2023 for the obtained sequences. The sequences were confirmed to be monkeypox virus.

### Validation of the MPXV laboratory-developed test

**Fig. 1:**
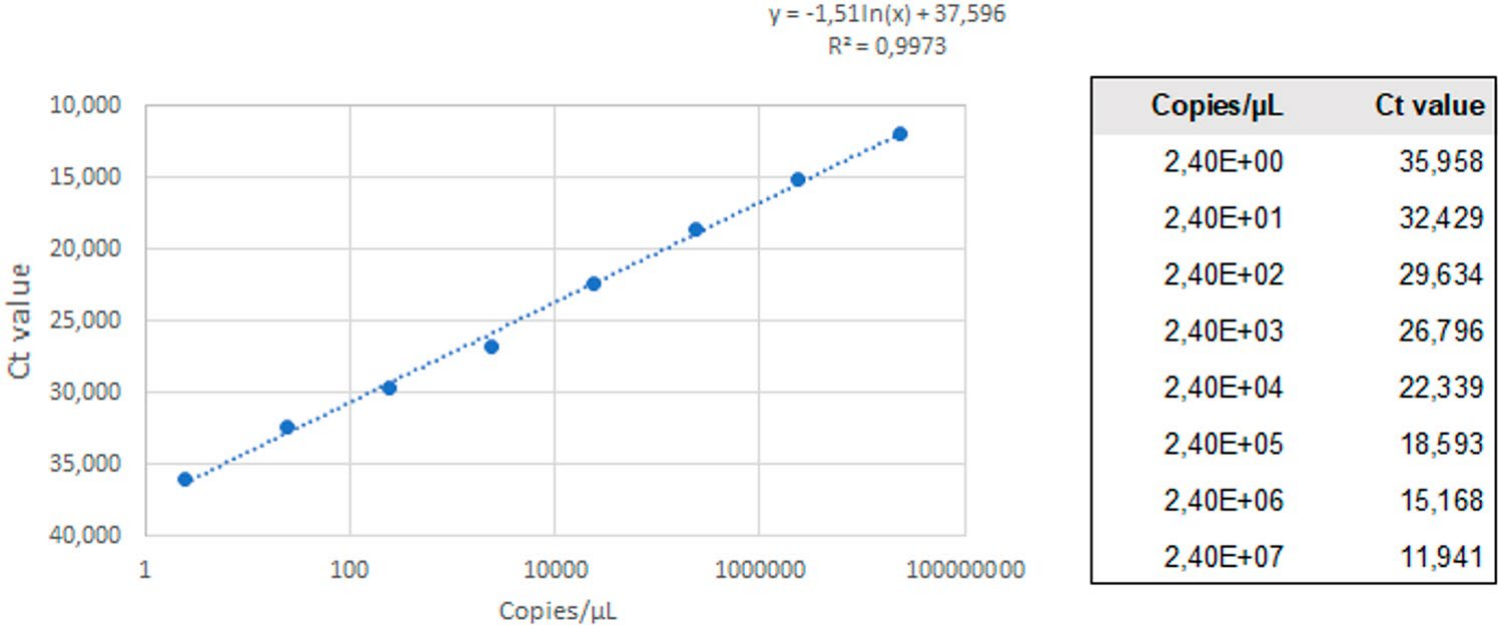
Standard curve LDT MPXV assay

A standard curve for copy number quantitation was generated by diluting the MPXV DNA standard (Integrated DNA Technologies, Belgium) to a tenfold dilution with concentrations between 1 and 1,000,000 copies per µL and determining the cycle threshold value (Ct) associated with each concentration. This was done in duplicate, and the mean Ct values were plotted against the MPXV standard concentration to generate a standard curve. The equation of the standard curve and the R2 value was determined by Excel version 2208 (Microsoft, Redmond, WA). The estimated number of detected copies of MPXV was extrapolated from the equation for the standard curve. The limit of detection was determined by testing 21 replicates of twofold dilutions containing MPXV copy numbers between 2.4 copies/µL and 0.3 copies/µL (Fig. 1).

To assess the sensitivity and specificity of the LDT, we tested 62 MPXV-positive samples (clade II) and 54 MPXV-negative samples (of which 16 VZV-positive and 2 HSV-2-positive samples) collected during the 2022 multi-country mpox outbreak (clade II) and compared it with the commercial TaqMan™ Monkeypox Virus Microbe Detection Assay (Applied Biosystems, Cat A50137), further referred to as TaqMan™ Monkeypox Assay. The LDT was performed with TaqMan Fast Virus 1-Step Master Mix (Applied Biosystems, USA) in a 20-µL reaction mixture containing 5-µL DNA template, 5-µL 4× TaqMan Fast Virus 1-Step Master Mix, 250-nM (FC) probe, 900-nM (FC) forward primer, 900-nM (FC) reverse primer, and water (Sigma-Aldrich, USA) up to 20 µL. The reactions were performed under the following conditions: 20 s at 95 °C and 45 cycles of 3 s at 95 °C and 30 s at 60 °C (Fast Mode). The reactions were run on a Quantstudio™ 7 Flex Real-Time PCR system and the analysis was done using Quantstudio Real-Time PCR Software, v1.7.2. (Applied Biosystems, Thermo Fisher). The commercial TaqMan™ Monkeypox Assay was conducted according to the manufacturer’s instructions on a Quantstudio™ 7 Flex Real-Time PCR system (Applied Biosystems, Thermo Fisher).

The LDT was also tested on five MPXV DNA samples from the DRC (clade I) in 2017. The reaction was performed in a 20-µL reaction mixture containing 5-µL DNA template, 5-µL 4× TaqMan Fast Virus 1-Step Master Mix, 250-nM (FC) probe, 900-nM (FC) forward primer, 900-nM (FC) reverse primer, and water (Sigma-Aldrich, USA) up to 20 µL. The reactions were performed under the following conditions: 20 s at 95 °C and 45 cycles of 3 s at 95 °C and 30 s at 60 °C (Fast Mode). The reactions were run on a 7500 Fast Real-Time PCR System (Applied Biosystems, USA) and analysis was done using the 7500 Fast System Software v1.3.1. The reactions could not be re-run on Quantstudio™ 7 Flex Real-Time PCR system due to the lack of DNA sample.

## Results

The BLAST search of the forward and reserve primers resulted in 100% query coverage and identity against vast majority of the sequences (> 99.5%). A single mismatch in the probe (ACCGGTAATCTTGTCGATGAGGACA) was found against the genomes of the West African clade, including those from the current outbreak.

The analytical sensitivity, i.e., the limit of detection as per the standard curve (Fig. 1), of our LDT was 1.2 copies/µL, with 95.5% of the replicates testing positive with a standard deviation of 0.972.

61 out of the 62 MPXV samples tested positive with the LDT (Table 2) with Ct spanning between Ct 13 and Ct 38. 30.7% had a Ct value lower than 20, which corresponds with over 115,000 copies/µL. 19.4% of the samples had a Ct value higher than 30 which corresponds with less than 153 copies/µL. The sample which tested negative with LDT showed a weak-positive result (Ct 38) with the TaqMan™ Monkeypox Assay. One MPXV sample also tested negative with TaqMan™ Monkeypox Assay, while it was confirmed positive with the LDT with a Ct of 35.9. The other 61 samples tested positive with the TaqMan™ Monkeypox Assay with Ct spanning between 13.6 and 38. The corresponding number of copies per µL cannot be determined for the commercial assay because the manufacturer does not disclose the ORF targeted by the assay and absolute quantification cannot be conducted. Median difference in Ct values between the LDT and the TaqMan™ Monkeypox Assay was 0.7 with the LDT showing lower Ct values. The results showed excellent concordance (98.3%) between the two assays. Diagnostic sensitivity of the LDT was determined at 99.14%. Diagnostic sensitivity of the TaqMan™ Monkeypox Assay is not reported by the manufacturer, while we determined it to be the same as our LDT at 99.14%.

**Table 2:** Performance of the LDT and TaqMan™ Monkeypox Assay

	Known MPXV positive (62)	Known MPXV negative (54)
LDT (tested MPXV positive)	61	0
LDT (tested MPXV negative)	0	54

	LDT sensitivity 99.14%	LDT specificity 100%
TaqMan™ Monkeypox Assay (tested MPXV positive)	61	0
TaqMan™ Monkeypox Assay (tested MPXV negative)	0	54
	TaqMan™ Monkeypox Assay sensitivity 99.14%	TaqMan™ Monkeypox Assay specificity 100%

All MPXV-negative samples (VZV and HSV2 positive) tested negative with both the LDT and the TaqMan™ Monkeypox Assay (Table 2) demonstrating 100% specificity.

**Fig. 2:**
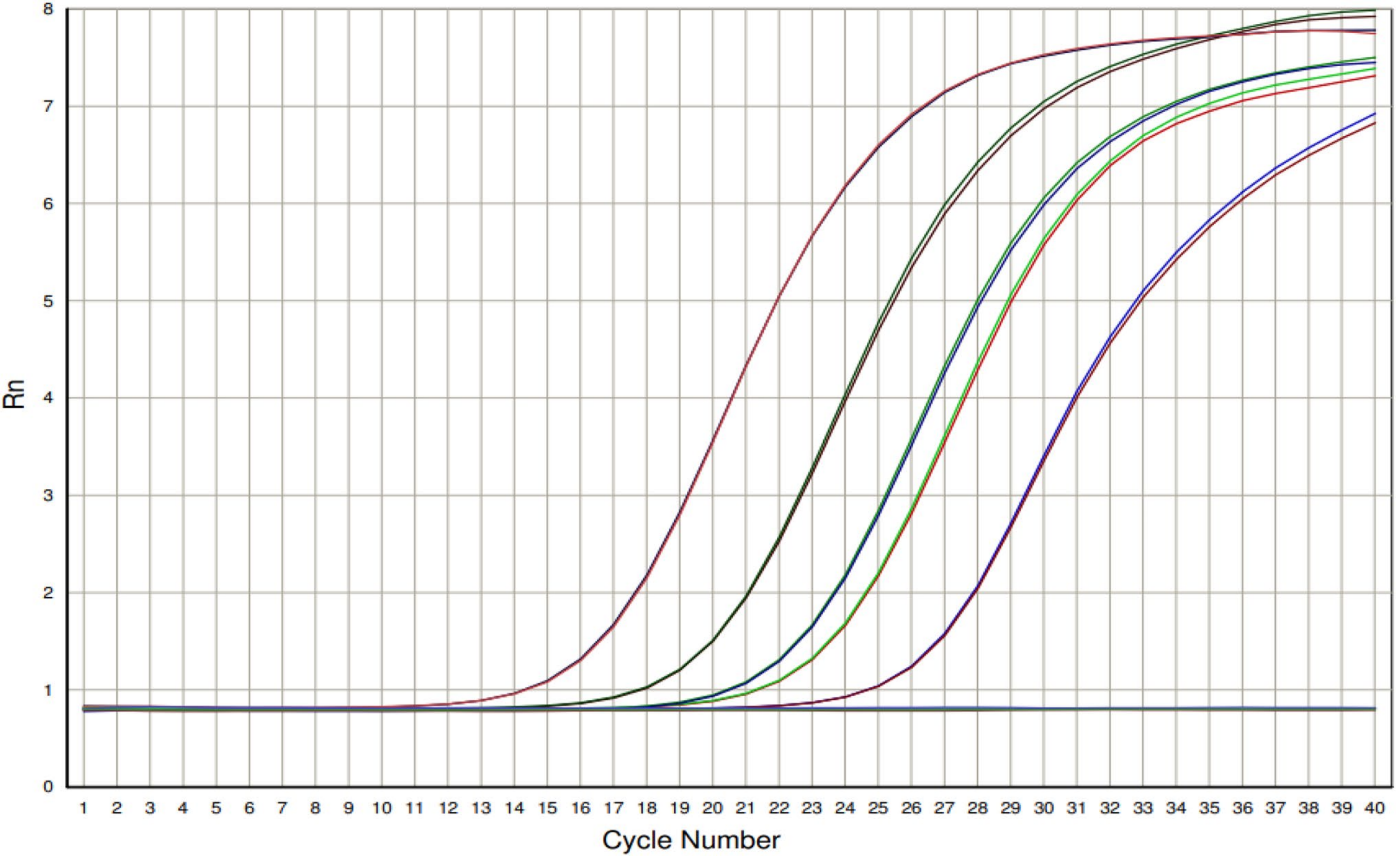
∆Rn vs cycle representation of the 5 MPXV clade I clinical samples using the LDT MPXV assay

All five MPXV DNA samples from the DRC were positive with Ct ranging from 15.3 to 24.6 (Fig. 2).

## Discussion

The multi-country outbreak of mpox which started in May 2022 played an important role in the development and manufacturing of commercial laboratory test for the diagnosis of mpox; however, their availability is not yet homogeneous across geographical regions. Commercial diagnostic assays are important especially in the routine mpox diagnosis in national reference laboratories. Nevertheless, there are instances where a reliable LDT might be appropriate to use, e.g., research purposes, shortage of commercial tests, transparency of primers and probe sequence, and ORF target, etc. We developed and validated an in-house real-time PCR assay and compared its performance with the commercial TaqMan™ Monkeypox Assay.

The LDT is able to detect both clades of the MPXV. Although we were able to investigate the sensitivity and specificity of the assay only against the clade II viruses from the current outbreak in Belgium, we confirmed in silico (against all known MPXV genomes) and on five clinical samples from the DRC that the assay is also able to detect clade I viruses. The minor genetic changes identified in the open reading frame O2L in some of the MPXV genomes did not impact the performance of our LDT. The manufacturer of the commercial MPXV assay reports they also conducted in silico analysis which showed 100% homology in 96% of the sequences included in their analysis and 4% of sequences having a mismatch at the 5′ end of a primer. They claim that this mismatch is insignificant and is not expected to impact performance [11]. However, we were not able to replicate the results because the manufacturer does not disclose the targeted viral ORF.

A warning was issued by the US Centers for Disease Control and Prevention concerning significant deletion in the tumor necrosis factor (TNF) receptor gene which is the target for some West African MPXV and generic MPXV real-time PCR tests. This deletion led to false-negative result with these tests [12]. Our LDT does not target the MPXV TNF receptor gene and deletion in this gene does not impact our LDT and its sensitivity. Whether the deletion does impact the TaqMan™ Monkeypox Assay cannot be determined because the manufacturer does not disclose information on the viral ORF targeted by the assay.

The analytical sensitivity of the LDT is 1.2 copies/µL which is lower than some other tested commercial assays and published LDTs [13, 14], and we were able to detect clinical samples with 3 copies/µL making the assay clinically relevant. The diagnostic sensitivity and specificity of the LDT was 99.14% and 100%, respectively. Out of 116 samples, only two MPXV samples showed discordance between our LDT and the commercial TaqMan™ Monkeypox Assay. One of those samples tested weak positive on the LDT with a Ct value of 35.9 which corresponds with 3.07 copies/µL and negative on the TaqMan™ Monkeypox Assay. The other sample had an even higher Ct value of 37.9 on the TaqMan™ Monkeypox Assay but tested negative on the LDT. This viral load is around the limit of detection of our LDT. Out of the 54 MPXV-negative samples, both assays tested 100% of the samples as negative.

The fact that all the samples (including the VZV and HSV-2) used for this MPXV LDT validation were collected as part of the current mpox outbreak upon a suspicion of mpox infection (with the same procedure for sample collection) warrants a representativeness of the LDT’s sensitivity and specificity in the context of a mpox outbreak.

The main limitation of the study is the inability to define sensitivity and specificity of the LDT on the clade I samples due to the limited number of samples available. Nevertheless, in silico evaluation and successful identification of the pathogen on 5 out of 5 Clade I DNA samples confirmed that the LDT can correctly identify both clades currently circulating worldwide. Additionally, specificity against other orthopoxviruses could not be validated due to lack of clinical samples. While variola is eradicated, cowpox and vaccinia are able to cause disease in humans but are not common.

## Data Availability

Data are contained within the article.
